# First installation of a dual-room IVR-CT system in the emergency room

**DOI:** 10.1186/s13049-018-0484-3

**Published:** 2018-03-05

**Authors:** Daiki Wada, Yasushi Nakamori, Shuji Kanayama, Shuhei Maruyama, Masahiro Kawada, Hiromu Iwamura, Koichi Hayakawa, Fukuki Saito, Yasuyuki Kuwagata

**Affiliations:** 1 Department of Emergency and Critical Care Medicine, Kansai Medical University Medical Center, 10-15 Fumizono-cho, Moriguchi, Osaka, 570-8507 Japan; 2 Department of Emergency and Critical Care Medicine, Kansai Medical University Hospital, 2-3-1 Shinmachi, Hirakata, Osaka, 573-1191 Japan

**Keywords:** Whole-body computed tomography, IVR-CT, Hybrid ER, Dual-room IVR-CT

## Abstract

Computed tomography (CT) embedded in the emergency room has gained importance in the early diagnostic phase of trauma care. In 2011, we implemented a new trauma workflow concept with a sliding CT scanner system with interventional radiology features (IVR-CT) that allows CT examination and emergency therapeutic intervention without relocating the patient, which we call the Hybrid emergency room (Hybrid ER). In the Hybrid ER, all life-saving procedures, CT examination, damage control surgery, and transcatheter arterial embolisation can be performed on the same table. Although the trauma workflow realized in the Hybrid ER may improve mortality in severe trauma, the Hybrid ER can potentially affect the efficacy of other in/outpatient diagnostic workflow because one room is occupied by one severely injured patient undergoing both emergency trauma care and CT scanning for long periods. In July 2017, we implemented a new trauma workflow concept with a dual-room sliding CT scanner system with interventional radiology features (dual-room IVR-CT) to increase patient throughput. When we perform emergency surgery or interventional radiology for a severely injured or ill patient in the Hybrid ER, the sliding CT scanner moves to the adjacent CT suite, and we can perform CT scanning of another in/outpatient. We believe that dual-room IVR-CT can contribute to the improvement of both the survival of severely injured or ill patients and patient throughput.

## Background

### Knowledge on whole-body computed tomography for trauma

Whole-body computed tomography (WBCT) has gained importance in the early diagnostic phase of trauma care. Huber-Wagner et al. [1] and Yeguiayan et al. [2] have suggested benefits of WBCT for patient survival. Although a multicentre randomised study by Sierink et al. showed that diagnosing patients with an immediate total-body CT scan does not reduce in-hospital mortality compared with the standard radiological work-up, they suggested that improvement in the selection of patients who may benefit from immediate total-body CT should be the subject of future research [3]. We have shown that CT performed before emergency bleeding control might be associated with improved survival, especially in severe trauma patients with a Trauma and Injury Severity Score probability of survival of < 50% [4]. Huber-Wagner et al. also showed that WBCT during trauma resuscitation significantly increased survival in haemodynamically unstable patients with major trauma [5].

### CT scanner installation within the emergency room

CT installed in the emergency room (ER) has also become important for early diagnosis in trauma care. Several studies have shown time and patient benefits in ER management by installing a CT gantry [6–8]. Although this concept substantially diminishes delays in transferring patients to the CT scanner, patient transfer to specialised departments for definitive therapy remain as one rate-limiting step in achieving maximum therapeutic workup.

### Introduction of IVR-CT into trauma workflow

**Fig. 1:**
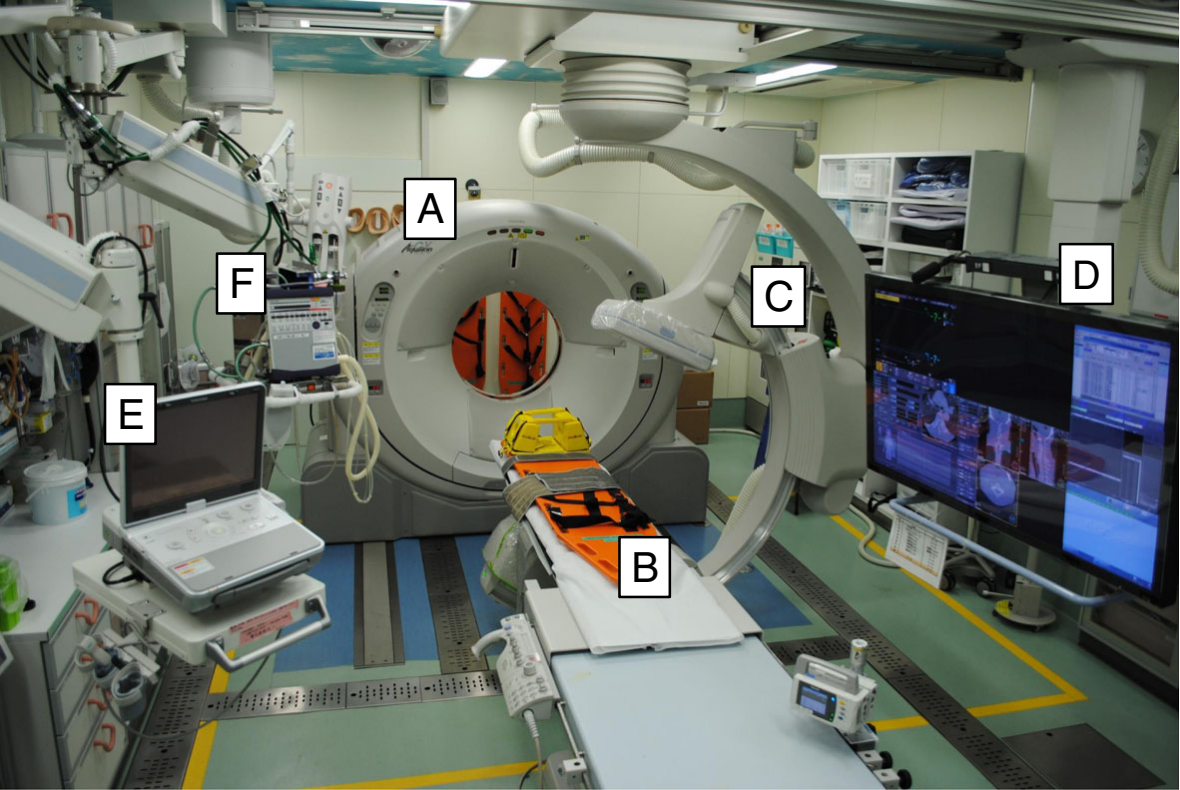
Photograph showing our IVR-CT system in the emergency room. All life-saving procedures including airway management, emergency surgery, and TAE can be performed on the table without relocating the patient. (A) Sliding CT scanner, (B) CT examination and intervention table, (C) moveable C-arm, (D) 56-in. monitor screen, (E) ultrasonography equipment, and (F) mechanical ventilator

We initially implemented a new trauma workflow concept with a sliding CT scanner system with interventional radiology features (IVR-CT) that allows CT examination and emergency therapeutic intervention without relocating the patient, which we call the Hybrid ER (Fig. 1). All workup times including time to CT initiation and start of bleeding control procedures (transcatheter arterial embolisation [TAE] and definitive surgical therapy) with our new protocol were significantly shorter than those with the conventional protocol [9]. Presently, eight trauma centres in Japan have a Hybrid ER. We previously revealed by retrospective cohort study at a tertiary hospital that this novel trauma workflow, comprising immediate CT diagnosis and rapid bleeding control without patient transfer, may improve mortality in severe trauma [10].

### Introduction of dual-room IVR-CT into trauma workflow

Being a one-room solution, the Hybrid ER can potentially affect the efficacy of other in/outpatient diagnostic workflow because one room is occupied by one severely injured patient undergoing both emergency trauma care and CT scanning. Recently, Frellesen et al. reported that sliding-gantry CT embedded in a dual-room solution without IVR features allows for significant time savings in the diagnostic workup of polytrauma patients, and faster resumption of the regular in/outpatient CT schedule is possible [11]. Therefore, in July 2017, we implemented a new trauma workflow concept with a dual-room IVR-CT to increase patient throughput.

## Main text

Before the introduction of the dual-room IVR-CT in our Hybrid ER trauma workflow concept, a multislice IVR-CT system (Aquilion PRIME, TSX-303B; Toshiba Medical Systems Corp., Tochigi, Japan) was installed in our ER. In our Hybrid ER workflow, when a patient is admitted to our ER, all life-saving procedures including airway management, CT examinations, emergency surgery, and TAE can be performed on the same table (Fig. 1). The primary advantage of our Hybrid ER workflow is the ability to initiate emergency bleeding control procedures after completion of the diagnostic workup without transferring the patient to the radiology department or operating room. The obvious disadvantage of this workflow is that the Hybrid ER can be occupied for long periods during bleeding control procedures.

Approximately 2000 patients are admitted to our emergency department per year. In 2016, we treated 380 (20%) trauma patients, which included 120 patients with severe trauma (Injury Severity Score > 16) and 1370 (73%) patients with severe illnesses such as sepsis, cardiovascular disease, cerebrovascular disease, gastrointestinal disease, and cardiopulmonary arrest. We have recently needed to cope with the diversifying needs of emergency medicine in Japan because of the important problem of elderly emergency patients as Japan’s society ages.

At that time, we had two conventional resuscitation rooms and the Hybrid ER. The Hybrid ER is available to both severely injured patients requiring definitive therapy and to severely ill patients such as those with acute myocardial infarction requiring percutaneous coronary intervention supported by veno-arterial extracorporeal membrane oxygenation (ECMO) or with acute interstitial pneumonia and acute respiratory failure requiring veno-veno ECMO. However, because such procedures occupy the Hybrid ER for long periods, we cannot perform CT examination in the Hybrid ER. This resulted in an economic problem in that the efficacy of other in/outpatient diagnostic workflow was reduced when the Hybrid ER was occupied.

**Fig. 2:**
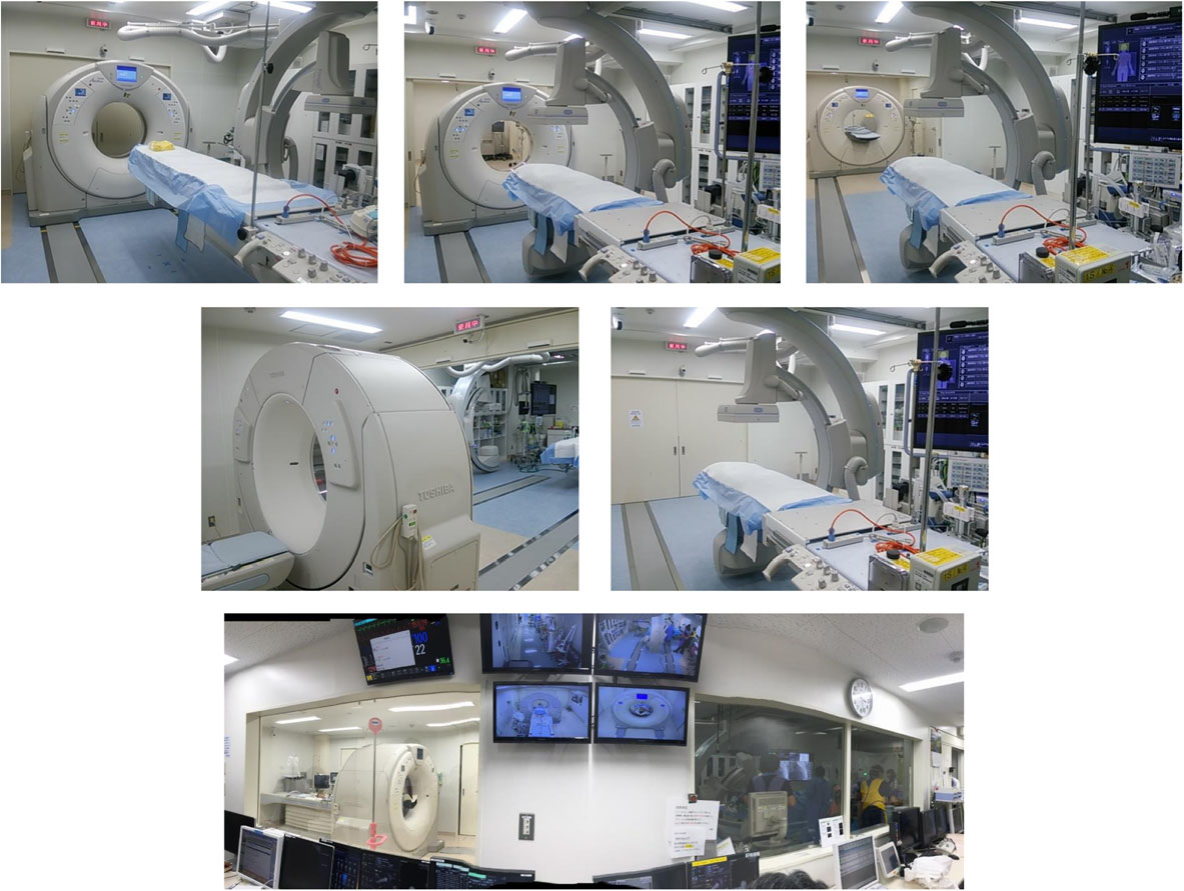
Photographs showing the dual-room sliding CT scanner system with interventional radiology features. The new CT suite has another radiolucent table. When we perform emergency surgery or interventional radiology for a severely injured or ill patient in the regular emergency room, the sliding CT scanner is moved to the new adjacent CT suite with the radiolucent table, and we can perform CT scanning of another in/outpatient

In July 2017, we implemented a new workflow concept with a dual-room IVR-CT. We installed one Hybrid ER and one new CT suite that has a radiolucent table (Fig. 2). These two rooms are separated by moveable door, and the sliding CT scanner moves between these two rooms depending on need. When we perform emergency surgery or IVR procedures for a severely injured or ill patient in the Hybrid ER, the sliding CT scanner is moved to the new CT suite, and we can perform CT scanning of another in/outpatient. Actually, while one severely injured patient is being treated in the existing Hybrid ER, if another moderately severe patient is admitted, the patient can be brought into the new CT suite for CT examination. After a diagnosis is made, the diagnostic room is rapidly vacated and readied for further use. If no patients are admitted to the Hybrid ER, intensive care unit patients scheduled for CT can be also brought into the new CT suite and scanned. To further reveal the efficiency of our system, we need to collect and evaluate the number of admitted in/outpatients and definitive interventions performed, especially of patients undergoing CT scans. There are several limitations in this study. First, this is a retrospective historical control study and not a randomized control study. Second, this study is conducted in a single institution. Third, the sample size is small. Thus, further study is required to evaluate the value of this workflow concept, including adverse events such as wound infection or sepsis that could be related to the performance of surgical procedures in the ER. We believe that this system may have a positive impact on the survival of severe trauma and seriously ill patients and improve patient throughput. In particular, we would like to see this system introduced to major trauma and tertiary care hospitals in urban areas and to local core hospitals in rural areas that treat trauma and seriously ill patients along with many moderately ill patients. However, there may be important limitations in terms of the 24-h availability of trauma surgeons, radiologists, and radiology technicians to take advantage of this system.

## Conclusion

We report a new workflow concept using a dual-room sliding CT scanner system with interventional radiology features. We believe that this system can contribute to improving the survival of severely injured and ill patients and can become the norm for patient throughput in emergency and critical care centres in the future.

## Abbreviations

ER: Emergency room
IVR: Interventional radiology
dual-room IVR-CT: Dual-room sliding CT scanner system with interventional radiology features
TAE: Transcatheter arterial embolisation
WBCT: Whole-body computed tomography

## References

[1] Huber-Wagner S, Lefering R, Qvick LM, Körner M, Kay MV, Pfeifer KJ (2009). Effect of whole-body CT during trauma resuscitation on survival: a retrospective, multicentre study. Lancet.

[2] Yeguiayan JM, Yap A, Freysz M, Garrigue D, Jacquot C, Martin C (2012). Impact of whole-body computed tomography on mortality and surgical management of severe blunt trauma. Crit Care.

[3] Sierink JC, Treskes K, Edwards MJ, Beuker BJ, Hartog D, Hohmann J (2016). Immediate total-body CT scanning versus conventional imaging and selective CT scanning in patients with severe trauma (REACT-2): a randomised controlled trial. Lancet.

[4] Wada D, Nakamori Y, Yamakawa K, Yoshikawa Y, Kiguchi T, Tasaki O (2013). Impact on survival of whole-body computed tomography before emergency bleeding control in patients with severe blunt trauma. Crit Care.

[5] Huber-Wagner S, Biberthaler P, Häberle S, Wierer M, Dobritz M, Rummeny E (2013). Whole-body CT in haemodynamically unstable severely injured patients--a retrospective, multicentre study. PLoS One.

[6] Hilbert P, Zur Nieden K, Hofmann GO, Hoeller I, Koch R, Stuttmann R (2007). New aspects in the emergency room management of critically injured patients: a multi-slice CT-oriented care algorithm. Injury.

[7] Fung Kon Jin PH, Gostlings JC, Ponsen KJ, van Kuijk C, Hoogerwerf N, Luitse JS (2008). Assessment of a new trauma workflow concept implementing a sliding CT scanner in the trauma room: the effect on workup times. J Trauma.

[8] Wurmb TE, Frühwald P, Hopfner W, Keil T, Kredel M, Brederlau J (2009). Whole-body multislice computed tomography as the first line diagnostic tool in patients with multiple injuries: the focus on time. J Trauma.

[9] Wada D, Nakamori Y, Yamakawa K, Fujimi S (2012). First clinical experience with IVR-CT system in the emergency room: positive impact on trauma workflow. Scand J Trauma Resusc Emerg Med.

[10] Kinoshita T, Yamakawa K, Matsuda H, Yoshikawa Y, Wada D, Hamasaki T, et al. The survival benefit of a novel trauma workflow that includes immediate whole-body computed tomography, surgery, and interventional radiology, all in one trauma resuscitation room: a retrospective historical control study. Ann Surg. 2017; 10.1097/SLA.0000000000002527. [Epub ahead of print] 10.1097/SLA.0000000000002527 PMC6325752 28953551

[11] Frellesen C, Boettcher M, Wichmann JL, Drieske M, Kerl JM, Lehnert T (2015). Evaluation of a dual-room sliding gantry CT concept for workflow optimisation in polytrauma and regular in- and outpatient management. Eur J Radiol.

